# Is embryo abortion a post‐zygotic barrier to gene flow between *Littorina* ecotypes?

**DOI:** 10.1111/jeb.13570

**Published:** 2019-12-07

**Authors:** Kerstin Johannesson, Zuzanna Zagrodzka, Rui Faria, Anja Marie Westram, Roger K. Butlin

**Affiliations:** ^1^ Department of Marine Sciences at Tjärnö University of Gothenburg Strömstad Sweden; ^2^ Department of Animal and Plant Sciences University of Sheffield Sheffield UK; ^3^ IST Austria Klosterneuburg Austria; ^4^ CIBIO Centro de Investigação em Biodiversidade e Recursos Genéticos InBIO Laboratório Associado Universidade do Porto Vairão Portugal

**Keywords:** Dobzhansky–Muller incompatibilities, gene flow barriers, maladaptation, malformed embryos

## Abstract

Genetic incompatibilities contribute to reproductive isolation between many diverging populations, but it is still unclear to what extent they play a role if divergence happens with gene flow. In contact zones between the "Crab" and "Wave" ecotypes of the snail *Littorina saxatilis,* divergent selection forms strong barriers to gene flow, while the role of post‐zygotic barriers due to selection against hybrids remains unclear. High embryo abortion rates in this species could indicate the presence of such barriers. Post‐zygotic barriers might include genetic incompatibilities (e.g. Dobzhansky–Muller incompatibilities) but also maladaptation, both expected to be most pronounced in contact zones. In addition, embryo abortion might reflect physiological stress on females and embryos independent of any genetic stress. We examined all embryos of >500 females sampled outside and inside contact zones of three populations in Sweden. Females' clutch size ranged from 0 to 1,011 embryos (mean 130 ± 123), and abortion rates varied between 0% and 100% (mean 12%). We described female genotypes by using a hybrid index based on hundreds of SNPs differentiated between ecotypes with which we characterized female genotypes. We also calculated female SNP heterozygosity and inversion karyotype. Clutch size did not vary with female hybrid index, and abortion rates were only weakly related to hybrid index in two sites but not at all in a third site. No additional variation in abortion rate was explained by female SNP heterozygosity, but increased female inversion heterozygosity added slightly to increased abortion. Our results show only weak and probably biologically insignificant post‐zygotic barriers contributing to ecotype divergence, and the high and variable abortion rates were marginally, if at all, explained by hybrid index of females.

## Graphical abstract

High abortion rates (12% in average) among females of the snail *Littorina saxatilis* might suggest postzygotic barriers to gene flow between ecotypes living in adjacent microhabitats. However, sampling several microhabitat transects did not more than marginally support increased rates of abortion in hybrid zones.

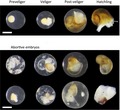

## 1 INTRODUCTION

Speciation involves the evolution and maintenance of reproductive isolation. This requires barriers that impede genetic exchange between populations (Coyne & Orr, 2004). Divergent selection, assortative mating and habitat selection will establish extrinsic barriers over smaller or larger parts of the genome (Jiggins & Mallet, 2000; MacCallum, Nurnberger, Barton, & Szymura, 1998). Barriers may also include intrinsic genetic elements, such as Dobzhansky–Muller incompatibilities (DMIs) which reduce fitness irrespective of environmental conditions. Also, partly intrinsic DMI‐analogous epistatic barriers might exist that are dependent on the environmental context (Arnegard et al., 2014; Kulmuni & Westram, 2017). Such intrinsic or partly intrinsic barriers (hereafter referred to as DMIs, Dobzhansky, 1937; Muller, 1942) are potentially important barriers that usually result from genetic divergence among populations during periods of isolation. At secondary contact, mixing of incompatible alleles at one or several loci will reduce hybrid fitness, and the effects are likely to be visible directly at the level of the phenotype as a viability or fertility reduction, either in first‐generation hybrids or in later generations of recombined offspring (Coyne & Orr, 2004; McDaniel, Willis, & Shaw, 2008).

Recent models and verbal arguments emphasize that DMIs can also evolve without prior isolation of populations, especially under strong local adaptation and divergent selection (Bank, Burger, & Hermisson, 2012; Gavrilets, 2004; Kondrashov, 2003; Kulmuni & Westram, 2017; Nosil & Flaxman, 2011). If inversions are involved, these form barriers to gene flow, and mutations suitable on the genetic background of one arrangement but deleterious on the background of the other may be established (Navarro & Barton, 2003). Such mutations will spread under positive selection, facilitated by recombination among haplotypes for the same arrangement (Faria, Johannesson, Butlin, & Westram, 2019b). However, when the two arrangements end up in the same heterokaryotype individual, which will most frequently happen in the hybrid zone, incompatibilities will be expressed.

Few studies have been undertaken to investigate the importance of intrinsic barriers when divergence evolves under gene flow, but the marine snail, *Littorina saxatilis*, presents an ideal model system. In this species, strong barriers to gene flow have evolved in contact zones between ecotypes, mainly by divergent selection (Johannesson et al., 2010), and evidence suggests that ecotypes repeatedly diverge without prior spatial isolation (Butlin et al., 2014; Panova, Hollander, & Johannesson, 2006). The divergent ecotypes differ in a range of independent, quantitative (and presumably polygenic) traits (reviewed in Johannesson, 2015), and this offers an excellent system to look for genetic incompatibilities or other types of maladaptation appearing in contact zones. Incompatibilities, if present, might have evolved as by‐products of strong divergent selection under gene flow (Kulmuni & Westram, 2017) and/or during short periods of allopatry (Bierne, Welch, Loire, Bonhomme, & David, 2011). Furthermore, several large and presumably old inversions segregate between ecotypes (Faria, Chaube, et al., 2019a; Morales et al., 2019; Westram et al., 2018) and these might have generated additional opportunities for DMIs to evolve as by‐products of divergent selection, or during earlier periods of isolation.

To test the importance of intrinsic barriers in *L. saxatilis,* we sampled females at precise positions over three transects covering pure ecotype populations and hybrid zones in between. The hybrid status of each female was assessed from a SNP‐based hybrid index, SNP heterozygosity and the inversion karyotype. Controlling for effects of external physiological stress (e.g. parasites and thermal stress), we tested the effect of female hybrid status on clutch size and embryo abortion rate. Our expectation was that intrinsic genetic incompatibilities (observed as smaller clutch size or higher abortion rates) would be most pronounced in genetic hybrids and less in females of parental genotype. In addition, we expected the DMI‐analogous barriers to be similar to those of classical DMIs, with the addition of a link to the environmental context (Arnegard et al., 2014). Furthermore, if present, decreased hybrid fitness due to heterozygote underdominance (incompatibility of alleles at the same locus) and/or maladaptation (lower fitness caused by a mismatch between genotype and environment) would also be more common in the hybrid zone than elsewhere. Consequently, at the phenotypic level, effects of underdominance and maladaptation would be difficult to separate from DMIs and a full distinction between intrinsic and extrinsic barriers would require later experimental approaches. However, our study was set out as a first step to test for an association between female hybrid status and abortion rates.

## 2 MATERIAL AND METHODS

### 2.1 Study system

Although several ecotypes of *Littorina saxatilis* exist (Reid, 1996), this study focused on Crab and Wave ecotypes that are adapted to specific rocky‐shore microhabitats (Johannesson, 2015; Johannesson et al., 2010). The Crab ecotype has a large adult size (10–15 mm), thick shell and a shy behaviour: traits that are favoured in patches with crabs. The Wave ecotype has a small adult size (3–7 mm), thin shell and a bold behaviour: traits that are favoured in wave‐exposed patches (Johannesson, 1986; Le Pennec et al., 2017). Contact zones are frequent, and over a Swedish contact zone divergent selection affects ~1.4% of SNPs (single nucleotide polymorphisms) over less than 50 metres, with most of the differentiated loci allocated to three large inversions which also contained many of the loci associated with the phenotypic differences (Westram et al., 2018). Notably, mark–recapture experiments at the same site suggested hybrid phenotypes to be superior to the parental phenotypes in the contact zone centre (Janson, 1983), thus showing no clear signs of hybrid maladaptation.

*Littorina saxatilis* is ovoviviparous with the very promiscuous females carrying broods of embryos year round (Panova et al., 2010). Phenotypic signals of post‐zygotic barriers can be followed during the entire early development, from fertilized eggs to metamorphosed juveniles, inside female's embryo pouches. Earlier studies have reported incongruent results with respect to rates of embryo abortion in females of various phenotypes (Hull, Grahame, & Mill, 1996; Janson, 1985; Johannesson, Larsson, Cruz, Garcia, & Rolán‐Alvarez, 2000). None of these earlier studies, however, classified females based on genotype, and a recent study in Spain showed that phenotypic hybrids are in many cases not genetic hybrids (Kess, Galindo, & Boulding, 2018).

### 2.2 Ecotype transects sampled

**Figure 1:**
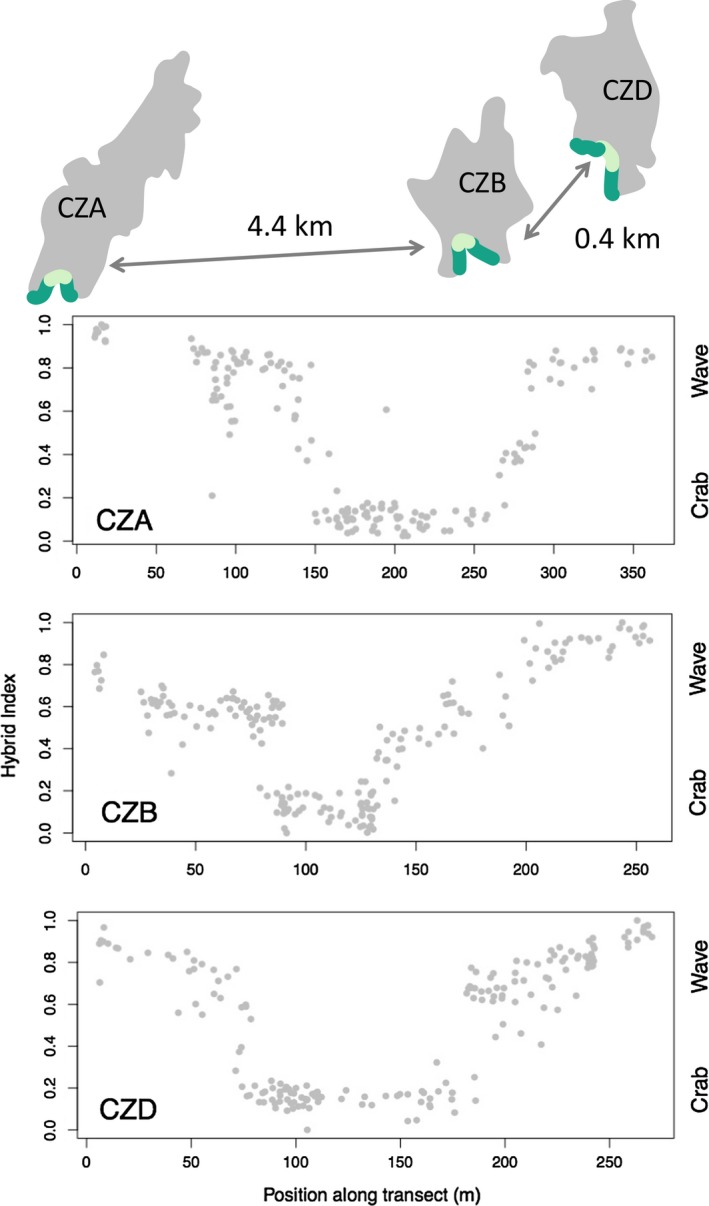
Along‐shore transects crossing habitats of Crab (light green) and Wave (dark green) ecotypes of *Littorina saxatilis* were sampled in three Swedish islands (here named CZA, CZB and CZD). Each transect included two Crab‐Wave contact zones (hybrid zones). Snail genotypes were characterized based on a large number of genetic markers and summarized in a hybrid index (see text for details) in each island normalized to range from 0 (extreme Crab genotype) to 1.0 (extreme Wave genotype). (Note that islands and distances are not to scale in the map)

We sampled 600 snails (of unknown sex) along 260–360 m long transects parallel to the shore in each of three islands (CZA; Ramsö N 58°49′27.90″, E 11°3′43.62″, CZB; Inre Arsklovet N 58°50′0.17″, E 11°8′19.08″ and CZD; Yttre Arsklovet N 58°49′51.38″, E 11°8′0.31″) during the period May–July, which is the peak reproductive season in this area. Each transect started on one side of a small bay in a Wave ecotype habitat (protruding wave‐exposed rocks; dark green in Figure 1 map), crossed a Crab ecotype habitat (a boulder bay with shore crabs; light green in Figure 1 map) and ended on the opposite side of the bay again on wave‐exposed rocks (Figure 1). Consequently, each transect crossed two Crab–Wave contact zones. The exact position of each snail was recorded in three dimensions using a Trimble M3 Total Station, including height on the shore. Maximum tidal variation is 35 cm in this area, and most snails were vertically distributed over <1 m with a few snails up to 2.5 m from the lowest record on the most wave‐exposed rocks. The embryos are likely to be affected by physiological stress, for example, extreme temperatures when the female is emerged above seawater. Here, we used the vertical position on the shore as a proxy for thermal stress (McMahon, 1990).

All snails were photographed for estimates of overall size and thereafter dissected with the soft tissue stored for genotyping (see below). The centroid size of each female was calculated from 15 landmarks placed on the shell photographs of each female (following Ravinet et al., 2016). The position of each female along the transect was referred to as its closest position on a path along the transect based on all sampled snails but reducing the spatial information of all sample points to a one‐dimensional transect.

### 2.3 Quantifying clutch size and embryo abortion

**Figure 2:**
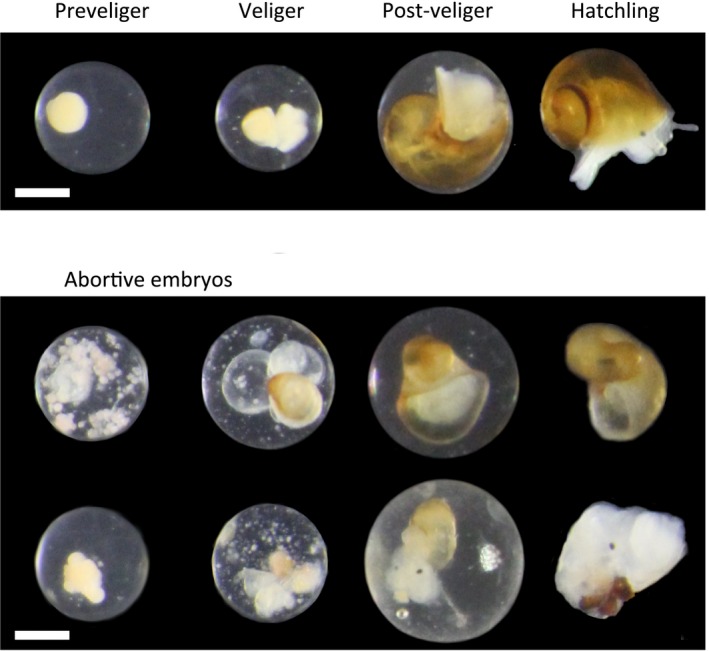
Normal and abortive embryos of four different developmental stages: preveliger (before development of swimming organs, vela), veliger (with vela), post‐veliger (after vela is shed), and hatchling (ready to leave the brood pouch). Abortive embryos have various types of severe developmental abnormalities and are presumably not able to survive outside the embryo pouch. The bar is 0.5 mm

The contents of the embryo pouch of each mature female (233, 219 and 217 females in the three islands, respectively) were emptied into a watch glass and photographed through the lens of a stereomicroscope at a magnification of 7–25×. We identified four larval stages using these photographs (following Janson, 1985): the preveliger stage (fertilized eggs up to veliger stadium), the veliger larva (a larva with two swimming organs—vela), the post‐veliger (the metamorphosed larva remaining inside the egg capsule) and the hatchling stage (a miniature snail ready to leave the mother) (Figure 2). For each larval stage, we also identified miss‐developing embryos (Figure 2, lower plate). These were typically clumps of cells spread throughout the capsule (miss‐developing preveligers), veligers and post‐veligers with malformed shells (trumpet‐like, poorly coiled, dwarfed, etc.). Miss‐developed embryos that were identified in this way were seriously affected and would rarely, if at all, actively leave the mother. Consequently, we refer to these as ‘abortive’ embryos. We counted numbers of normal and abortive embryos at each embryo stage for each female, and abortion rates (for each stage) were defined as the number of abortive embryos in that stage divided by the total number of embryos in the same stage. Importantly, it was the same person who did all the image analyses, and this person did not have any prior information about hybrid status or transect position of a female. The order of processing females was randomized. Females also sometimes had multiple embryos in the same egg capsule but these were overall few; however, they were counted separately if one was normal and the other malformed. A ciliate (presumably *Protophrya ovicola*) was commonly observed inside the embryo pouch of females. This ciliate is small but when present mostly occurred in >10× the number of embryos, moving over the surface of egg capsules. We considered the presence of ciliates as a potential source of physiological stress, and recorded the presence or absence of ciliates for all females. A few females (<1%) were infected by trematode parasites. These females were all sterile and without embryos, and they were not included in downstream analyses.

### 2.4 Characterizing female hybrid status

A random subset of the 600 sampled snails per site was genotyped using capture sequencing (Illumina sequencing based on 40,000 randomly distributed probes, see Westram et al., 2018 for details). This generated genotype information for 172, 192 and 180 of the analysed females from the three islands after appropriate filtering of the genetic data. The sequencing generated 69,386 SNP loci (methods as in Westram et al., 2018) from which a hybrid index (HI) was calculated by summing reads over loci for the reference allele and dividing by the sum of total reads across both alleles. The reference allele was defined as the allele that was more common in the Wave ecotype than the Crab ecotype, and loci were only included if the allele frequency difference between the Wave and Crab ends of the transect was >0.1. The hybrid index was then scaled to range from 0 (most extreme Crab genotype) to 1 (most extreme Wave genotype), separately for each island. Heterozygosity for each female was obtained by calling genotypes using a pipeline from Westram et al. (2018) and counting the proportion of loci for which the individual was heterozygous. For the final estimates, we used only loci outside the inversions identified by Faria, Chaube, et al. (2019a) and with an *F*_ST_ between ecotypes of at least 0.4 in either of the two contacts of a given island. The reason for this filtering was to avoid confounding general effects of heterozygosity (that we were looking for) with specific effects of polymorphic inversions (which we tested separately). Focusing on loci with high *F*_ST_ was to concentrate on the effects of admixture between ecotypes. Individuals that had genotype data for fewer than 50 SNPs after this filtering were excluded. We also considered the effect of genotype for each of five inversions LGC1.1, LGC2.1, LGC6.1/2, LGC14.1/2 and LGC17.1 (Faria, Chaube, et al., 2019a) that showed strong and consistent clines at the three study sites (A.M. Westram, R. Faria, K. Johannesson, R.K. Butlin, unpublished data). We calculated inversion heterozygosity as the proportion of the inversions that were heterokaryotypic in each female. One potential effect of inversions is that homokaryotypes suffer more from deleterious recessive alleles than heterokaryotypes and so females with low levels of inversion heterozygosity would have increased rates of embryo abortion. On the other hand, if the two different arrangements of an inversion (the inverted and the ancestral arrangements) had acquired incompatible alleles, we would see the opposite trend.

Genotyping of female *L. saxatilis* was primarily used to identify female hybrid status. Importantly, however, earlier results indicate that hybrid zones are populated by later generation hybrids and backcrosses rather than F1 and F2 generation hybrids (Westram et al., 2018). Furthermore, as both males and females usually only disperse 1–2 m during their life‐time (Westram et al., 2018), a female's genotype will in most cases also reflect the average hybrid status of the ~20 males that sired her brood of embryos (Panova et al., 2010), and consequently also the average hybrid status of the embryos.

### 2.5 Separating different factors

In order to test whether any genetic incompatibilities (or maladaptations) appearing in hybrid females tended to reduce the total number of embryos per female (clutch size), we needed to control for effects of female size, shore height (thermal stress) and presence/absence of ciliates in the brood pouch. We did this using generalized linear models (GLMs), with Poisson errors and log‐link function, and included female centroid size, shore height, presence/absence of ciliates and hybrid index in a 4‐factor analysis. We also tested a quadratic effect of the hybrid index since these effects should be greatest for intermediate values.

Next, we analysed any genetic incompatibilities (or maladaptations) in hybrid females affecting embryo abortion rates. We accounted for effects of clutch size and height on shore, in addition to presence/absence of ciliates, using a 4‐factor analysis of the number of abortive embryos as a proportion of the total number of embryos. These analyses were done separately for each stage of larval development using logistic regression, that is GLMs with binomial errors and logit link function. Because of very low numbers of miss‐developing individuals, we excluded the hatchlings from all analyses that were done separately for each larval stage. Again, we included quadratic effects, predicting maximum abortion at intermediate values for the hybrid index (i.e. close to the centre of the contact zone where HI = 0.5).

We expected (and observed, see below) a strong relationship between female heterozygosity and hybrid index. Therefore, we did not include heterozygosity in the models but we checked, after fitting other explanatory variables, whether it improved model fit. We expected linear relationships: reduced clutch size and higher abortion rate with increasing heterozygosity, because the most heterozygous individuals are expected to be early‐generation hybrids.

We used a similar approach for the statistical evaluation of effects of female inversion heterozygosity, calculated across all five inversions. We added this variable to the best models to test whether taking into account overall female inversion heterozygosity explained any additional component of variation in embryo abortion rate.

Initial exploratory analyses showed strong effects of island and interactions between island and the other explanatory variables. Therefore, we report analyses for each island separately. We chose models by starting with a full model and eliminating nonsignificant terms. Statistical significance was tested by comparing the change in deviance to a chi‐square distribution. Where we report statistical significance, we use the conventional criterion of α = 0.05 but we do not infer biological significance using this binary distinction. We observed over‐dispersion in most cases and so we also tested the deviance ratio (mean deviance explained/residual mean deviance) against an *F* distribution. These tests gave qualitatively similar results and so they are not reported. Analyses were conducted in R version 3.5.1.

## 3 RESULTS

**Figure 3:**
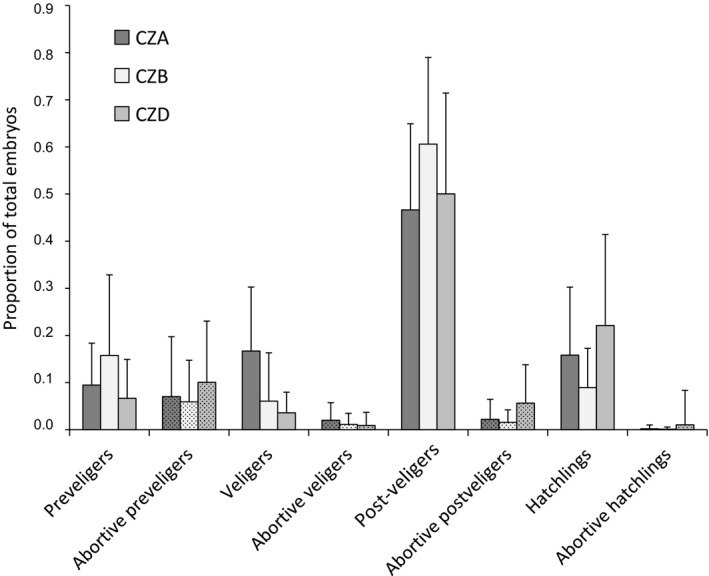
Average proportions of normally developing and abortive embryos of four different developmental stages (preveliger, veliger, post‐veliger and hatchling) in samples of female *L. saxatilis* from three different islands (CZA, CZB and CZD). Error bars indicate standard deviations

After removing females with fewer than 10 embryos (5% of all genotyped females), we ended up with 171, 192 and 180 genotyped females analysed in the islands CZA, CZB and CZD, respectively. On average, a female carried 105 ± 92 (stand. dev.) embryos in CZA, 159 ± 142 in CZB and 121 ± 121 in CZD with an overall maximum number of 1,011. The proportions of embryos in the different stages of development were similar among the three islands with 50%–60% of all embryos being in the post‐veliger stage and the rest roughly equally distributed over preveliger, veliger and hatchling stages (Figure 3).

The proportion of abortive embryos was very high among the preveligers with 27%–60% of the preveligers of a female being abortive, on average (Figure 3). The proportion of abortive embryos at later stages was much lower and lowest among hatchlings (Figure 3). Although population averages were similar, the frequency of abortive embryos varied widely among individual females in each island, ranging from 0% to 100% of the embryos per developmental stage (Figure S1).

The hybrid index analysis clearly showed that the shore transects passed through six contact zones in which individuals of hybrid genotype were present (Figure 1). The three islands differed somewhat in the proportion of hybrids encountered, with more females having intermediate hybrid index values in CZB than in CZA and CZD. The reason for this seemed to be a relatively shallow environmental transition in CZB between the crab and wave environments due, at least in part, to this transect being somewhat less exposed to the open sea.

### 3.1 Effect of hybrid index on clutch size

Our 4‐factor model explored effects of female size, ciliates, shore height and hybrid index on clutch size. We focus first on the three extrinsic factors. As expected, clutch size correlated strongly with size of female (Figure S2). Ciliates were common in brood pouches of Crab genotype females in all three islands with 64%, 38% and 56% of the Crab ecotype females (HI < 0.3) having ciliates in CZA, CZB and CZD, respectively. Ciliates were less common, or absent, in hybrid and Wave ecotype females (Figure S3). The presence of ciliates was, surprisingly, associated with increased clutch size in CZB and CZD, while showing the opposite trend in CZA (Table S1). Shore height, finally, showed lower clutch size up the shore, as expected from increased physiological stress with longer periods of being outside the water (Table S1).

Allowing for effects of female size, ciliates and shore height, clutch size peaked at intermediate values of the hybrid index in all three islands (Table S1), with peaks shifted towards the wave end of transects in CZA and CZD. Consequently, for a mean size female without ciliates, fitted values of clutch size were larger (CZA), or much larger (CZD), both for females with intermediate hybrid index (hybrids) and for females with high hybrid index (Wave ecotype), compared to females with low index (Crab ecotype) (Table S2). In CZB, clutch size peaked around HI = 0.5, that is among the hybrid females. Similar results were obtained if comparing clutch size excluding the abortive embryos (Table S2).

### 3.2 Effect of hybrid index on abortion rate

Our 4‐factor model to explore effects of shore height, ciliates, clutch size and female hybrid index on the rate of embryo abortion showed variable effects of the four factors. Before focusing on the effect of hybrid index, we first present the effect of the other factors. Shore height affected embryo abortion rate as either a linear, or a nonlinear, increase up the shore at all sites and in all stages except post‐veligers at CZB (Table S3) with doubled abortion rates in the preveliger stage, in all three islands, over a metre of shore height (Figure S4). Ciliates were associated with lower abortion rates in preveligers, veligers and post‐veligers in both CZA and CZD, and in preveligers in CZB (Table S3). On average, ciliate presence was associated with a reduction in abortive embryos by >30% (Table S4). In CZA, proportions of abortive embryos decreased for all three larval stages (preveliger, veliger and post‐veliger) with increased clutch size (Table S3). However, this effect was not seen in the other two islands.

**Figure 4:**
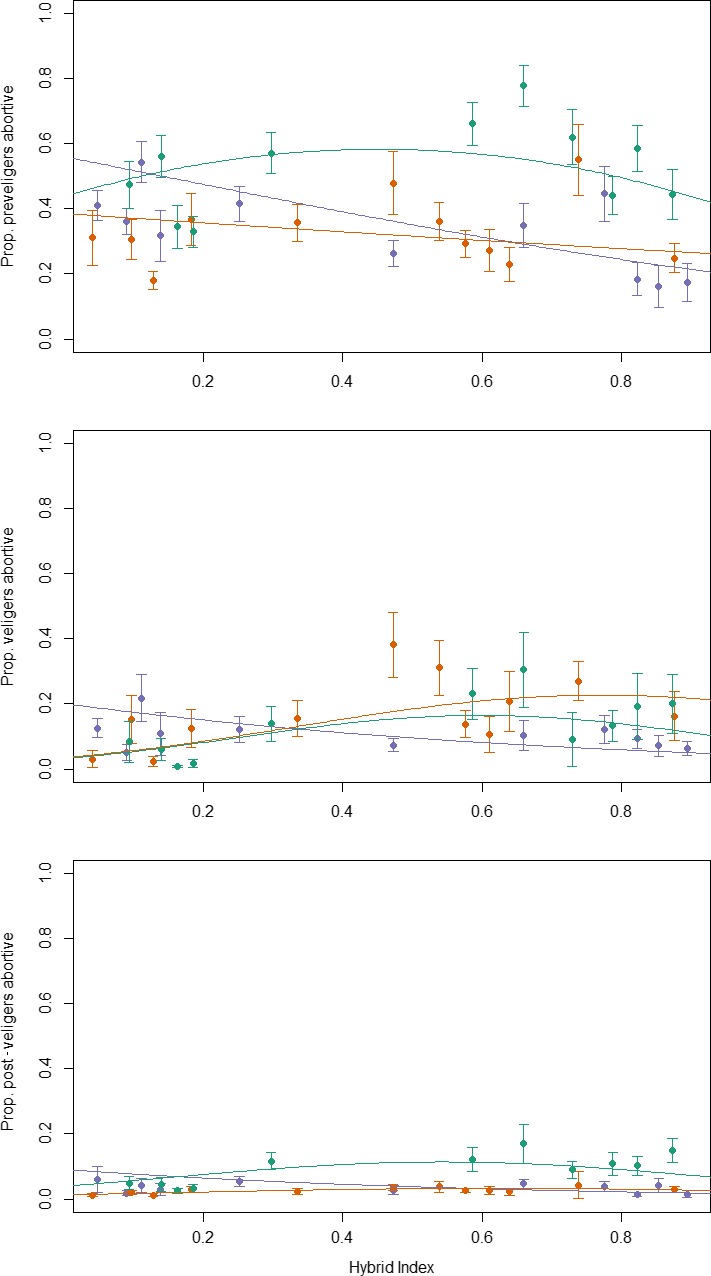
Proportions of abortive (misdeveloped) embryos in females of *L. saxatilis* sampled over transects from three different islands (mauve—CZA, orange—CZB and green—CZD) as a function of female hybrid index (see Figure 1 and text). Points are weighted means, ± standard error, for groups of 15 consecutive females ordered by hybrid index (within island) and plotted at their mean hybrid index. Lines are the linear or quadratic relationships with hybrid index from a model that also included effects of shore height, ciliate presence/absence and total embryos (where significant, details in Table S3)

After controlling for effects of the other three factors (shore height, ciliates and clutch size), we found no evidence for higher abortion rates in hybrid females in CZA. Instead, linear models gave the best fits to the data for all three larval stages with the highest proportions of embryo abortion in females of Crab ecotype (Table S3, Figure 4). In the other islands, quadratic models with peaks of embryo abortion at intermediate hybrid indexes gave the best fit to the data, but not among preveligers in CZB where abortion rates decreased with hybrid index. The peaks of highest abortion rates shifted slightly from close to a hybrid index of 0.5 for the preveliger stage in CZD towards the Wave ecotype for the veliger and post‐veliger stages in CZB and CZD (Figure 4). In total, our models explained 4.2%–35.8% of the variation in embryo abortion rates among females and this included the relatively strong effects of shore height and ciliates with hybrid index explaining <1%–19% of variation (Table S3). Therefore, we conclude that the effect of hybrid index was overall small and possibly of little biological consequence relative to the wide variation in abortion rates among females.

It is possible that location effects in our transects were not fully captured using the hybrid index, especially because a few females appeared as outliers on the hybrid index‐location plots (Figure 1). However, plotting the deviance residuals from the 4‐factor model above did not indicate any location effects associated with habitat boundaries (Figure S5).

### 3.3 Effects of SNP and inversion heterozygosity on abortion rate

As expected, levels of SNP heterozygosity peaked at intermediate values of the hybrid index in all three islands. However, variation in heterozygosity was large over the complete hybrid index range (Figure S6), probably due to the fact that the SNPs were not fixed differently in Crab and Wave ecotypes (Westram et al., 2018). As SNP heterozygosity and hybrid index were highly correlated, we did not enter both in the primary models. When adding SNP heterozygosity to the final model (Table S3), it did not improve the model further (data not shown).

When adding inversion heterozygosity to the final model, we observed peak embryo abortion rates at inversion heterozygosities around 0.29–0.62 in CZB and CZD, but no effects in CZA. As most inversion heterozygosity values were in the range 0.15–0.30, peak values represented the upper range of estimates, offering some further support for the potential presence of weak genetic incompatibilities in females with increased numbers of inversion heterokaryotypes in CZB and CZD. Although genotypes for individual inversions sometimes showed statistically significant effects, there was no consistent pattern (Table S4).

## 4 DISCUSSION

A strong candidate trait for a genetic incompatibility in *Littorina saxatilis* (or, less likely, a symptom of maladaptation) is embryo abortion, which is observed throughout the species' distribution (Sweden—Janson, 1985; White Sea—Sokolova, 1995; the UK—Johnson, Mill, Hull, & Ducrotoy, 2000; Spain—Johannesson et al., 2000). It seems unlikely that this is a purely additive genetic effect, as strong selection would immediately reduce the extreme variation present. Here, we investigated the relationship between embryo abortion and the female hybrid status (also reflecting the average hybrid status of her offspring) to test whether or not post‐zygotic barriers (potentially including genetic incompatibilities) contribute to the genetic barrier between the two ecotypes.

Earlier studies have shown that exposing females to extreme salinity, toxic substances, or similar, results in increased rates of embryo abortion (Dixon & Pollard, 1985; Sokolova, 1995; Sánchez‐Argüello, Aparicio, & Fernández, 2012, but see Clyne & Duffus, 1979). As expected, we found that abortion rates were influenced by female and so embryo environments. Increased physiological stress in the high intertidal (McMahon, 1990) most likely explains the increase in abortion rates up the shore (although genetic clines parallel to shore height are present in this species and might also contribute Johannesson, Johannesson, & Lundgren, 1995; Morales et al., 2019). Somewhat more surprising was the observation that ciliates, particularly common in the Crab ecotype in our study area (while Bojko, Grahame, & Dunn, 2017, found them to be more common in the Wave ecotype in UK), were present in increased numbers in females with lower numbers of abortive embryos, by up to 30%. The relationship between the ciliates and their hosts deserves further attention.

Although physiological stressors may affect gene expression and thereby cause developmental effects, ‘genetic stress’ caused by incompatible Crab‐Wave genotypes involved in epistatic interactions or heterozygote underdominance may give similar deleterious effects in hybrids. After correcting for the effects of ciliates, shore height and clutch size, we remained with weak peaks of abortion rates in female hybrid genotypes in CZB and CZD (with a nonsignificant effect in preveligers in CZB and no peak in CZA). Whether what we see from these analyses indicates biologically meaningful effects over longer periods of time is difficult to infer, but on a short time‐perspective, the addition to gene flow barriers from these post‐zygotic effects is likely to be negligible, whereas divergent selection and possibly assortment contribute to strong barriers to gene flow between Crab and Wave ecotypes (Johannesson et al., 2010; Morales et al., 2019; Westram et al., 2018). This situation is different from contact zones in many other species where barriers, even between closely related taxa, often show strong DMI effects (Barton & Hewitt, 1981; Coyne & Orr, 1989; Szymura & Barton, 1991; Turner & Harr, 2014). However, when strong DMIs are present, hybrid zones are typically secondary with genetic differences established during periods of isolation prior to formation of contact zones. In *L. saxatilis,* our previous data suggest primary origins of hybrid zones under gene flow (Butlin et al., 2014), and the evolution of genetic incompatibilities might be constrained by gene flow, unless it is unidirectional or weak (Bank et al., 2012; Gavrilets, 2004; Nosil & Flaxman, 2011). On the other hand, as most genes have multiple functions and are involved in epistatic interactions with many other genes, evolution of (relatively weak) DMIs should be common in any system where differential selection drives divergence, whether or not gene flow occurs (Kulmuni & Westram, 2017). DMI‐like epistatic effects may also interact with the environment, as shown in diverging ecotypes of stickleback, where reduction in fitness is due to mismatch of adaptive traits (Arnegard et al., 2014).

That female embryo abortion rate is also affected by the external environment (e.g. ciliates and shore height) is not surprising. However, we have observed that females raised in the laboratory remain with high and very variable levels of embryo abortion despite a homogeneous environment, continuous submergence and no ciliates (K. Johannesson, Personal observation). This suggests intrinsic factors probably remain important, even if in this study we have not been able to identify the factors that play a large role in explaining abortion rates. Perhaps, one reason is that, even if a genetic component of the variation in abortion rate among females is an epistatic side effect of divergent adaptation (and therefore not removed by selection), this effect might involve loci, for example, placed within different inversions and so incompatibilities will appear for embryos with certain combinations of chromosomal arrangements. If so, we are unable to identify this effect from genotyping the females; even though this tells us something about the average embryo genotype, single embryos of the same brood will carry different inversion karyotypes, in particular in the hybrid zone and under frequent multiple mating (Panova et al., 2010). Thus, we suggest that in a future study the genotypes of individual embryos should be assessed, and the genotypes of single embryos should be compared with the embryo's development.

## Supporting information


**Suppl. Table 1.** The effects of four factors: hybrid index, female centroid size, ciliates presence or absence and shore height on female clutch size.
**Suppl. Table 2.** Fitted values of total number of female embryos for low, medium and high hybrid index.
**Suppl. Table 3.** The effects of four factors: hybrid index, shore height, ciliate presence or absence, and total number of embryos per female on abortion rate at preveliger, veliger and postveliger stages of larval development.
**Suppl. Table 4.** Predicted proportions of aborting embryos at mean hybrid index, shore height and total number of embryos for different developmental stages.
**Suppl. Table 5.** Effects of inversions on abortion rates in females.
**Suppl. Fig. 1.** Variation in proportion of abortive embryos among females from Crab‐Wave transects.
**Suppl. Fig. 2.** Total number of embryos in females of different sizes.
**Suppl. Fig. 3.** Presence and absence of ciliates (Protophrya ovicola) in the embryo pouches of females of different hybrid index.
**Suppl. Fig. 4.** Variation in proportion of abortive embryos among females in relation to shore height.
**Suppl. Fig. 5.** Deviance residuals from the models in Supplement Table 3 in relation to transect location.
**Suppl. Fig. 6.** The relationship between heterozygosity and hybrid index

## Data Availability

Data deposited at Dryad: doi: https://doi.org/10.5061/dryad.tb2rbnzwk
