# Supplementary material for: Is embryo abortion a post‐zygotic barrier to gene flow between Littorina ecotypes?
Source: J Evol Biol. 2019 Dec 7;33(3):342–51. doi: 10.1111/jeb.13570 (PMC7079066; doi:10.1111/jeb.13570)

Supplementary information

"Is embryo abortion a postzygotic barrier to gene flow between *Littorina* ecotypes?"

Authors: Kerstin Johannesson, Zuzanna Zagrodzka, Rui Faria, Anja Marie Westram, Roger K Butlin

Suppl. Table 1. The effects of four factors: hybrid index, female centroid size, ciliates presence or absence and shore height on female clutch size for populations of *L. saxatilis* sampled over Wave-Crab ecotype transects crossing ecotype hybrid zones in three Swedish islands (CZA, CZB and CZD). Effect sizes are reported only for effects that remained after elimination of non-significant terms. *** indicates significance of the overall fit: $P < 0.001$. The percentage of total deviance explained provides an indication of model adequacy. df – degrees of freedom used for testing the deviance explained.

Sample	Explanatory variables	Best fit model	Pattern	Linear effect (± SE)	Quadratic effect (± SE)	Position of peak
Clutch size						
CZA	Hybrid index	Quadratic	Peak	0.752 ± 0.112	-0.562 ± 0.136	At HI=0.67
	Female size	Linear	Increase	0.167 ± 0.003	-	
	Ciliates		Decrease	-0.0657 ± 0.0160	-	
	Shore height	Qaudratic	Peak	0.0385 ± 0.0862	-0.229 ± 0.0605	Height = 0.084m
			Deviance explained:	7768*** (64.9%)	df =6	
CZB	Hybrid index	Quadratic	Peak	2.184 ± 0.089	-2.385 ± 0.095	At HI=0.46
	Female size	Quadratic	Leveling	0.237 ± 0.008	-0.00318 ± 0.00026	Above value range
	Ciliates		Increase	0.0796 ± 0.0166	-	
	Shore height	Quadratic	Decrease	-0.0795 ± 0.0939	-0.460 ± 0.0985	Below value range
			Deviance explained:	13703*** (68.2%)	df = 7	
CZD	Hybrid index	Quadratic	Peak	3.087 ± 0.139	-2.199 ± 0.146	At HI=0.70
	Female size	Quadratic	Leveling	0.368 ± 0.010	-0.00679 ± 0.00035	Above value range
	Ciliates		Increase	0.119 ± 0.017	-	
	Shore height	Linear	Decrease	-0.563 ± 0.0389		
			Deviance explained:	11317*** (65.8%)	df = 6	

Suppl. Table 2. Fitted values of total number of female embryos for low, medium and high hybrid index (HI) corresponding to Crab, hybrid and Wave ecotypes, respectively. Values were fitted for mean female size and absence of ciliates. Females of *Littorina saxatilis* were sampled along shore transects from Crab to Wave ecotype habitats including hybrid zones in three Swedish islands (CZA, CZB and CZD).

	**HI=0.1**	**HI=0.5**	**HI=0.9**
Total number of embryos including abortive			
CZA	72.1	85.1	83.9
CZB	118.0	159.4	100.4
CZD	69.3	140.6	141.0
Total number of embryos excluding abortive			
CZA	60.8	75.2	77.9
CZB	113.6	142.8	88.6
CZD	58.3	113.2	118.1

Suppl. Table 3. The effects of four factors: hybrid index, shore height, ciliate presence or absence, and total number of embryos per female on abortion rate at preveliger, veliger and postveliger stages of larval development for populations of *L. saxatilis* sampled over Wave-Crab ecotype transects crossing ecotype hybrid zones in three Swedish islands (CZA, CZB and CZD). Effect sizes are reported only for effects that remained after elimination of non-significant terms. *** indicates significance of the overall fit: P < 0.001. The percentage of total deviance explained provides an indication of model adequacy. df – degrees of freedom used for testing the deviance explained. We also give the percentage of deviance explained specifically by the hybrid index.

Sample	Explanatory variables	Best fit model	Pattern	Linear effect (± SE)	Quadratic effect (± SE)	Position of peak (dip)
Preveliger abortion rate						
CZA	Hybrid index	Linear	Decrease	-1.72 ± 0.179	-	
	Shore height	Quadratic	Dip	-275.7 ± 139.9	0.693 ± 0.350	Below value range
	Ciliates	Linear	Decrease	-0.654 ± 0.0884	-	
	Clutch size	Linear	Decrease	-0.00193 ± 0.000386	-	
			Deviance explained:	139.6*** (Total: 19.7%, HI: 4.5%)	df=5	
CZB	Hybrid index	Linear	Decrease	-0.611 ± 0.161	-	
	Shore height	Quadratic	Peak	1150 ± 211	-2.90 ± 0.53	Above value range
	Ciliates	Linear	Decrease	-0.592 ± 0.0907	-	
	Clutch size	Linear	Increase	0.000984 ± 0.000156	-	
			Deviance explained:	161.0*** (Total: 8.5%, HI: <1%)	df=5	

CZD	Hybrid index	Quadratic	Peak	2.60 ± 0.686	-2.88 ± 0.721	At HI=0.45
	Shore height	Quadratic	Peak	680 ± 155	-1.71 ± 0.389	At lower part of shore
	Ciliates	Linear	Decrease	-0.646 ± 0.0968	-	
	Clutch size	Linear	Increase	0.000518 ± 0.000238	-	
			Deviance explained:	258.1*** (Total: 25.2%, HI: 6.2%)	df=6	

Veliger abortion rate

CZA	Hybrid index	Linear	Decrease	-1.74 ± 0.260	-	
	Shore height	Linear	Increase	1.12 ± 0.212	-	
	Ciliates	Linear	Decrease	-0.830 ± 0.127	-	
	Clutch size	Linear	Decrease	-0.00190 ± 0.00059	-	
			Deviance explained:	90.3*** (Total: 13.0%, HI: 1.1%)	df=4	

CZB	Hybrid index	Quadratic	Peak	5.42 ± 0.95	-3.51 ± 0.91	At HI=0.77
	Shore height	Linear	Increase	1.29 ± 0.31	-	
	Clutch size	Linear	Increase	0.00245 ± 0.00030	-	
			Deviance explained:	188*** (Total: 22.7%: HI: 13.5%)	df=4	

CZD	Hybrid index	Quadratic	Peak	5.99 ± 2.09	-5.00 ± 2.04	At HI=0.60
	Shore height	Quadratic	Peak	3316 ± 1166	-8.34 ± 2.94	At lower part of shore
	Ciliates	Linear	Decrease	-0.912 ± 0.370	-	
			Deviance explained:	155*** (Total: 35.8%, HI: 14.6%)	df=5	

Postveliger abortion rate

CZA	Hybrid index	Linear	Decrease	-1.87 ± 0.233	-	
	Shore height	Quadratic	Peak	312 ± 130	-0.779 ± 0.324	Below value range
	Ciliates	Linear	Decrease	-1.06 ± 0.117	-	
	Clutch size	Linear	Decrease	-0.00381 ± 0.000574	-	
			Deviance explained:	153*** (Total: 18.6%, HI: <1%)	df=5	

CZB	Hybrid index	Quadratic	Peak	3.04 ± 0.661	-2.43 ± 0.719	At HI=0.63
			Deviance explained:	35.9*** (Total: 4.2%, HI: 4.2%)	df=2	

CZD	Hybrid index	Quadratic	Peak	4.19 ± 0.609	-3.86 ± 0.602	At HI=0.54
	Shore height	Linear	Increase	2.03 ± 0.154	-	
	Ciliates	Linear	Decrease	-0.864 ± 0.108	-	
			Deviance explained:	600*** (Total: 34.9%, HI: 19.0%)	df=4	

Suppl. Table 4. Predicted proportions of aborting embryos at mean hybrid index, shore height and total number of embryos for different developmental stages (preveliger, veliger and postveliger) in three Swedish islands (CZA, CZB and CZD).

**Stage**	**Island**	**No ciliates (A)**	**Ciliates (B)**	**B/A**
Preveliger	CZA	0.360	0.226	63%
	CZB	0.344	0.251	73%
	CZD	0.704	0.554	79%
Veliger	CZA	0.054	0.024	44%
	CZB	0.138	0.138	100%
	CZD	0.408	0.217	53%
Postveliger	CZA	0.011	0.004	36%
	CZB	0.026	0.026	100%
	CZD	0.169	0.079	47%
			Overall	*66%*

Suppl. Table 5. Effects of inversions on abortion rates in females of *L. saxatilis* in three different islands. Top section of each table describes a quadratic fit for inversion heterozygosity and peak/trough values are indicated. Lower section describes fitted effects for the homozygote genotypes at each inversion in relation to the heterozygote (higher or lower proportion of abortive embryos) after accounting for effects of female hybrid index, shore height, female total number of embryos and brood pouch ciliates (presence/absence). Only significant effects are shown (deviance change for 2df, $p<0.01$).

Preveliger abortion

	**Island CZA**			**Island CZB**			**Island CZD**		
	Additional deviance explained	Peak/Trough	Peak position	Additional deviance explained	Peak/Trough	Peak position	Additional deviance explained	Peak/Trough	Peak position
Inversion heterozygosity	-	-	-	10.88	Peak	0.34	11.68	Peak	0.34
Inversion:		Homokary. 1	Homokary. 2		Homokary. 1	Homokary. 2		Homokary. 1	Homokary. 2
LGC1.1	16.26	↓	↓	27.11	↑	↑	10.99	↑	-
LGC2.1	13.71	↓	↑	34.97	↓	-	12.98	↓	↓
LGC6.1	-	-	-	18.72	↓	↓	29.55	↑	↓
LGC14.1	19.65	↓	↓	-	-	-	23.26	↓	↑
LGC17.1	22.28	↑	↑	44.62	↓	↓	12.71	-	↓

Veliger abortion

	Island CZA			Island CZB			Island CZD		
	Additional deviance explained	Peak/Trough	Peak position	Additional deviance explained	Peak/Trough	Peak position	Additional deviance explained	Peak/Trough	Peak position
Inversion heterozygosity	-	-	-	11.46	Peak	0.29	-	-	-
Inversion:		Homokary. 1	Homokary. 2		Homokary. 1	Homokary. 2		Homokary. 1	Homokary. 2
LGC1.1	-	-	-	-	-	-	-	-	-
LGC2.1	-	-	-	24.01	↑	↑	12.59	-	↓
LGC6.1	-	-	-	47.4	↓	-	-	-	-
LGC14.1	-	-	-	15.69	↓	↑	-	-	-
LGC17.1	-	-	-	-	-	-	-	-	-

Postveliger abortion

	**Island CZA**			**Island CZB**			**Island CZD**		
	Additional deviance explained	Peak/Trough	Peak position	Additional deviance explained	Peak/Trough	Peak position	Additional deviance explained	Peak/Trough	Peak position
Inversion heterozygosity	-	-	-	-	-	-	48.33	Peak	0.62
Inversion:		Homokary. 1	Homokary. 2		Homokary. 1	Homokary. 2		Homokary. 1	Homokary. 2
LGC1.1	24.23	↑	-	-	-	-	10.62	-	↓
LGC2.1	-	-	-	-	-	-	-	-	-
LGC6.1	-	-	-	-	-	-	-	-	-
LGC14.1	20.38	↑	↓	-	-	-	-	-	-
LGC17.1	12.69	↓	↓	-	-	-	15.58	↓	↓

Suppl. Fig. 1. Variation in proportion of abortive (miss-developed) embryos among all females sampled in Crab-Wave transects (hybrid index 0 is Crab end and 1.0 is Wave end of transects) in three Swedish islands (point colour mauve-CZA, orange-CZB, and green-CZD). Notice that for all different developmental stages (preveliger, veliger and postveliger) there are single females that have only misdeveloped embryos, but also many females that have no visible signs of abortive embryos. Point sizes are scaled by the logarithm of the total number of embryos of each female (range 10 – 1000).

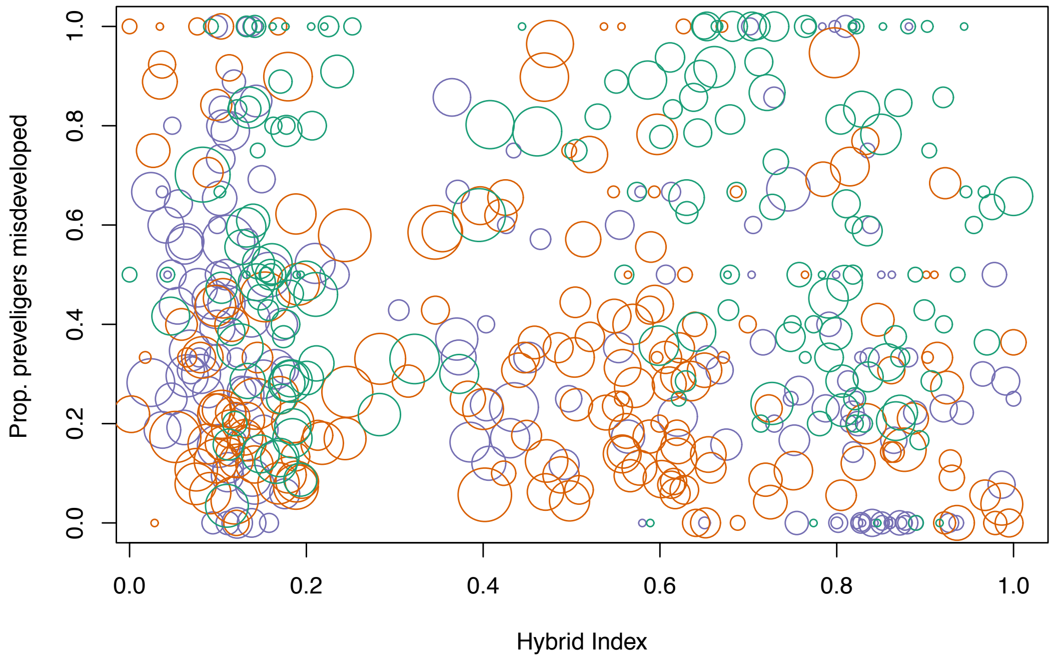

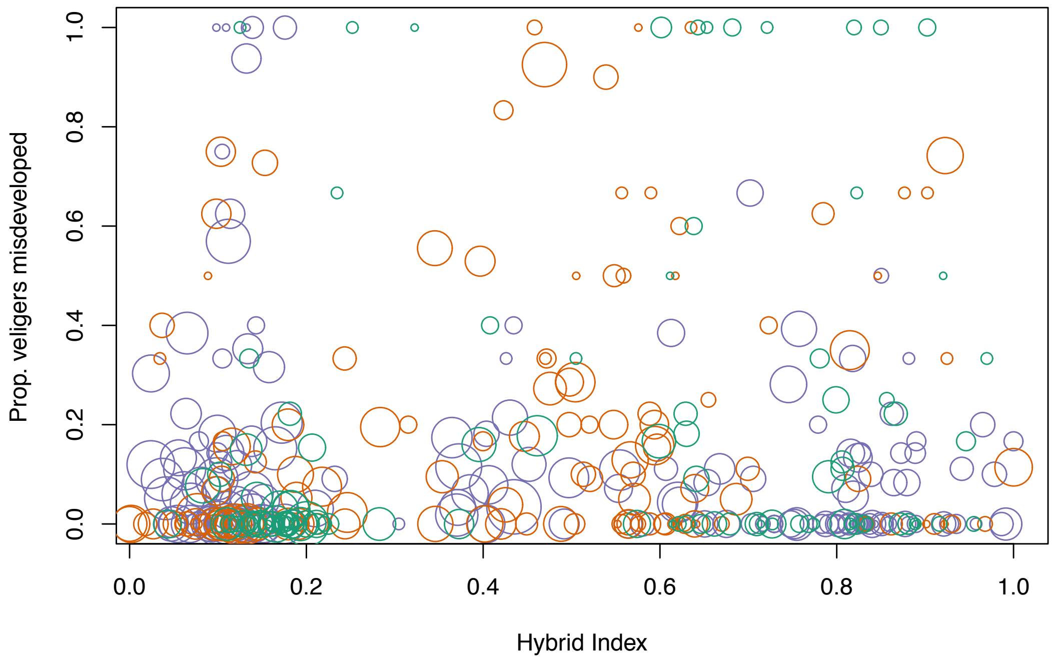
Prop. veligers misdeveloped
0.0
0.2
0.4
0.6
0.8
1.0
Hybrid Index

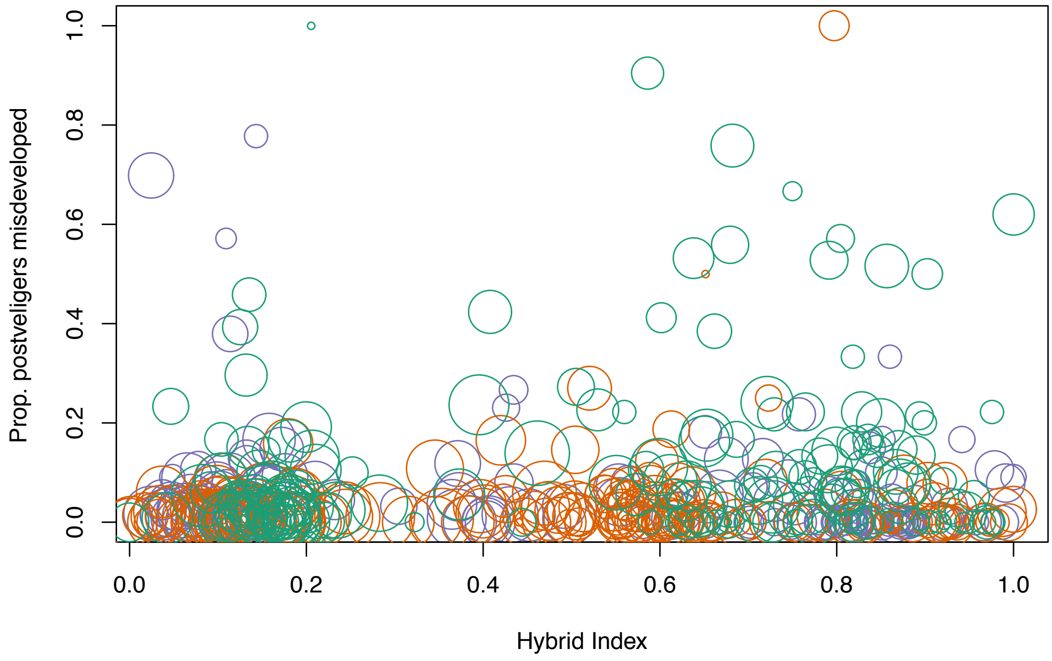
Prop. postveligers misdeveloped
0.0
0.2
0.4
0.6
0.8
1.0
Hybrid Index

Suppl. Fig. 2. Total number of embryos in females of *L. saxatilis* of different sizes (measured as centroid size) from three Swedish islands (mauve-CZA, orange-CZB, and green-CZD). Grey squares are females with hybrid index >0.7 (Wave ecotype end of spectrum), open circles are females of intermediate hybrid index, and grey triangles are females with hybrid index <0.3 (Crab ecotype end of spectrum).

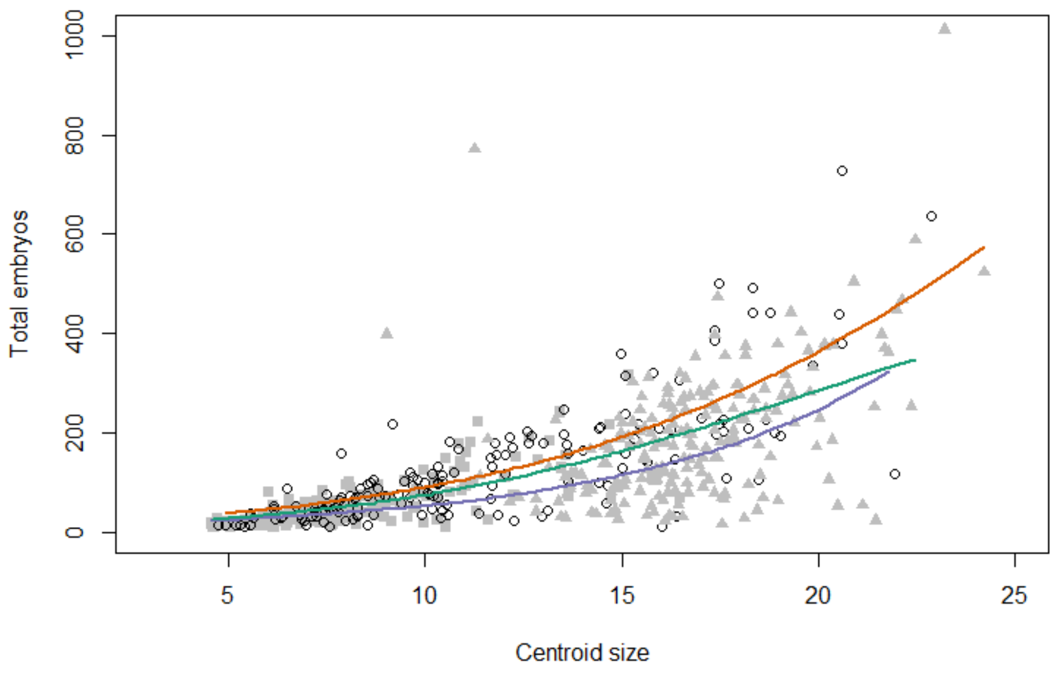

Suppl. Fig. 3. Presence and absence of ciliates (*Protophrya ovicola*) in the embryo pouches of female *L. saxatilis* of different hybrid index (see Fig. 1 and text) in three Swedish islands (CZA, CZB and CZD). A low hybrid index indicates Crab ecotype and a high index Wave ecotype. The box plots show the median hybrid index for each group, the upper and lower quartiles and the range. The violins show the smoothed density distribution.

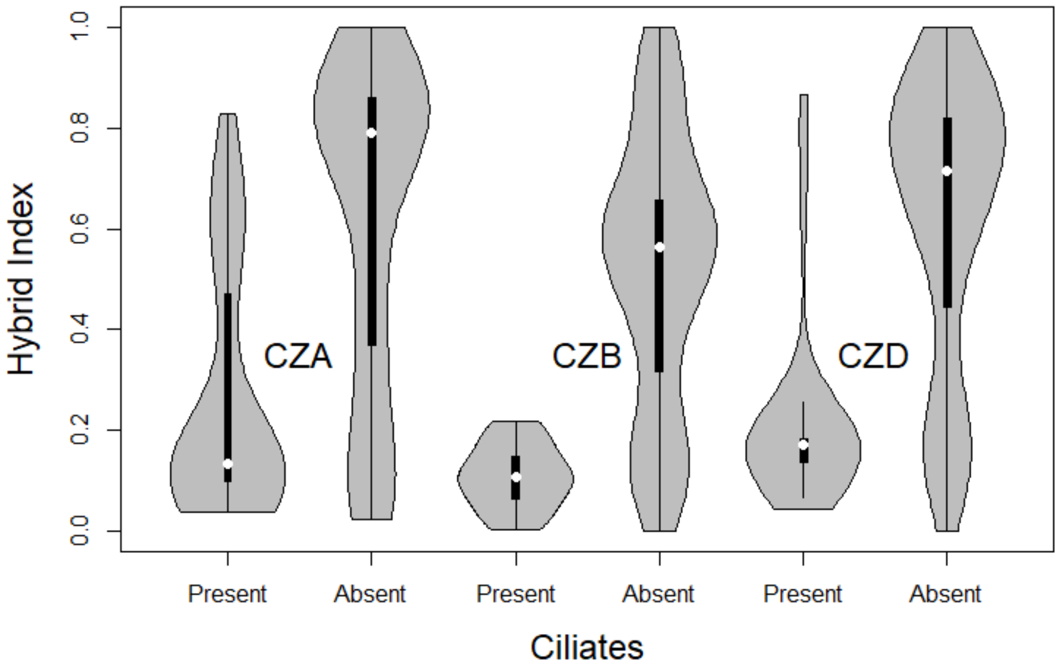

Suppl. Fig. 4. Variation in proportion of abortive (miss-developed) embryos among all females sampled in three Swedish islands (point colour mauve-CZA, orange-CZB, and green-CZD) in relation to shore height (metres above lowest recorded snail) where a female was sampled. The illustrations show trends of increasing rates of miss-development from low to high shore level. Point sizes are scaled by the logarithm of the total number of embryos of each female (range 10 – 1000). Fitted lines are expectations at the means for other variables (hybrid index, female size, ciliates and total number of embryos).

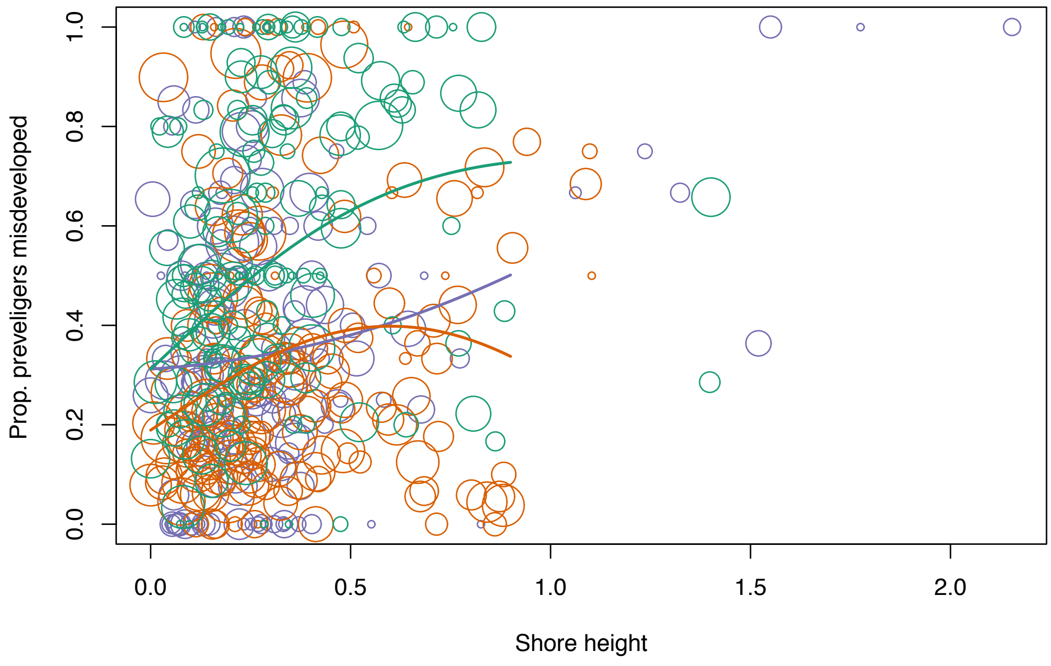

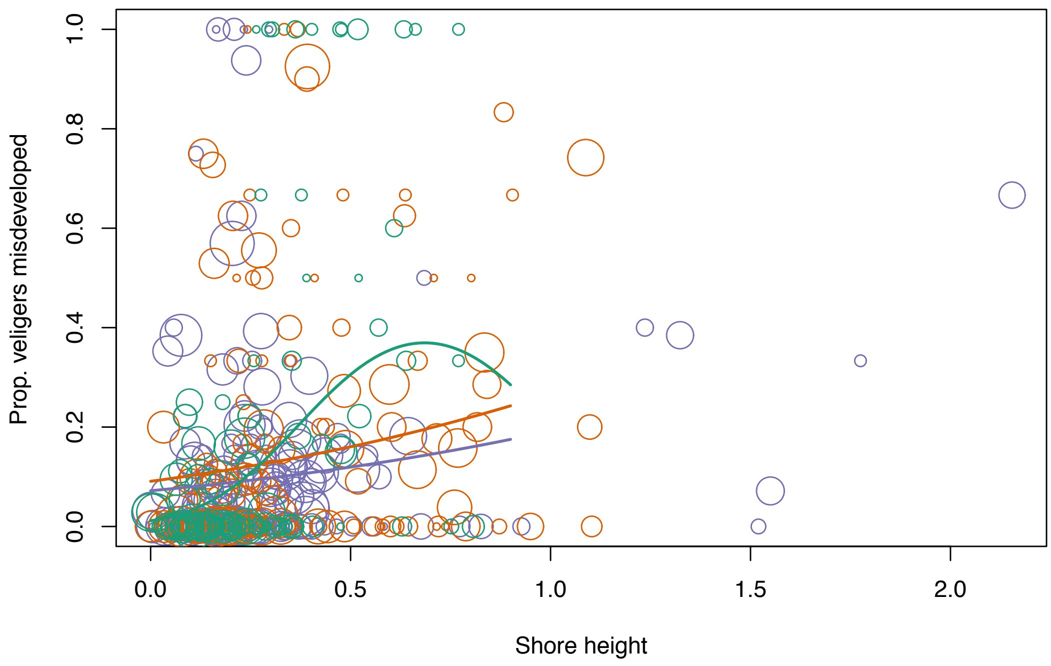
Prop. veligers misdeveloped
0.0
0.2
0.4
0.6
0.8
1.0
0.0
0.5
1.0
1.5
2.0
Shore height

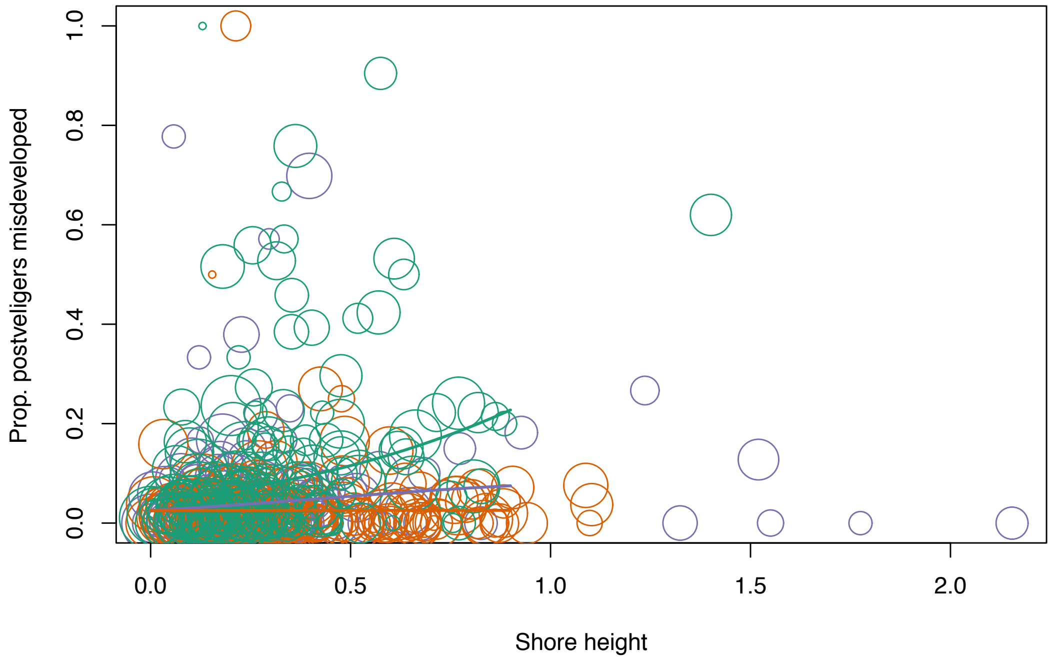
Prop. postveligers misdeveloped
0.0
0.2
0.4
0.6
0.8
1.0
0.0
0.5
1.0
1.5
2.0
Shore height

Suppl. Fig. 5. Deviance residuals from the models in Supplement Table 3 in relation to transect location. The models included the effects of female hybrid index, shore height, ciliate presence/absence and clutch size on abortion rate. Each larval developmental stage and island is plotted separately. See Figure 1 for positions of contact zones. Plots do not suggest any additional effect of the interaction between female hybrid index and position along the transect.

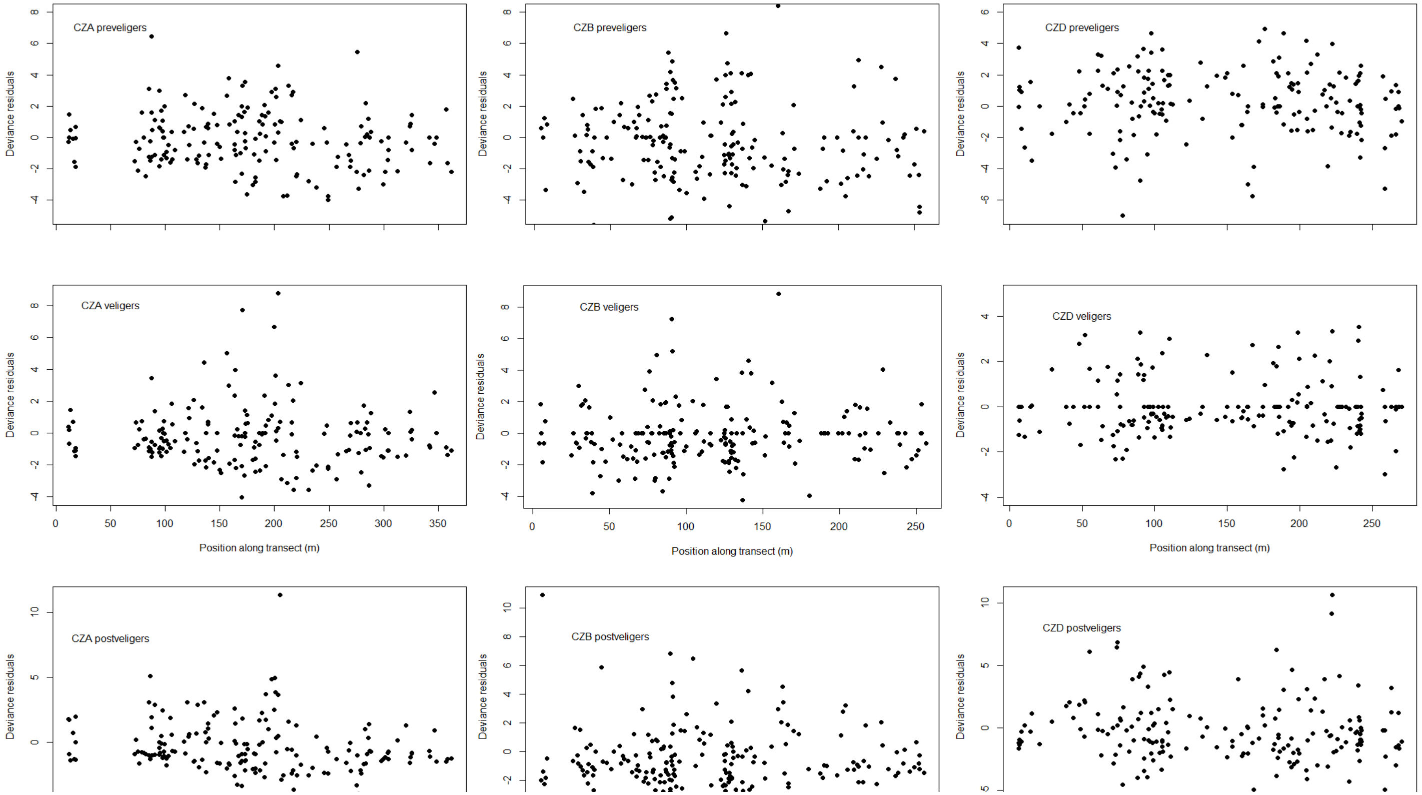

Suppl. Fig. 6. The relationship between heterozygosity and hybrid index (see text for details) in females of *L. saxatilis* from transects covering ecotype hybrid zones in three different islands (mauve-CZA, orange-CZB, and green-CZD).

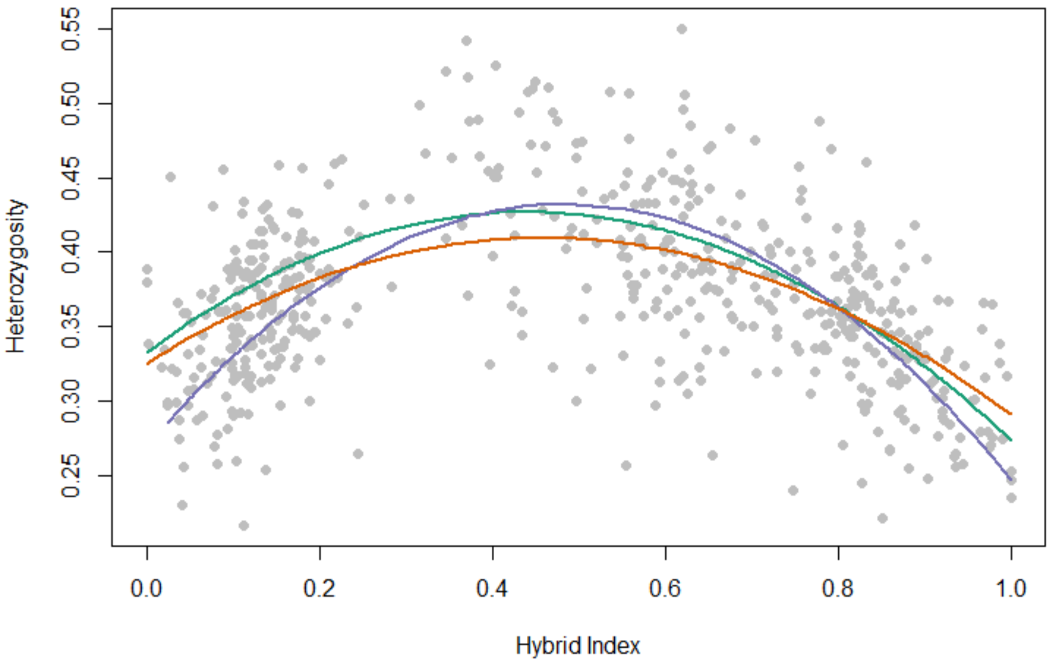

Supplement: Supplementary file 1 — Suppl. Table 1. The effects of four factors: hybrid index, female centroid size, ciliates presence or absence and shore height on female clutch size. Suppl. Table 2. Fitted values of total number of female embryos for low, medium and high hybrid index. Suppl. Table 3. The effects of four factors: hybrid index, shore height, ciliate presence or absence, and total number of embryos per female on abortion rate at preveliger, veliger and postveliger stages of larval development. Suppl. Table 4. Predicted proportions of aborting embryos at mean hybrid index, shore height and total number of embryos for different developmental stages. Suppl. Table 5. Effects of inversions on abortion rates in females. Suppl. Fig. 1. Variation in proportion of abortive embryos among females from Crab‐Wave transects. Suppl. Fig. 2. Total number of embryos in females of different sizes. Suppl. Fig. 3. Presence and absence of ciliates (Protophrya ovicola) in the embryo pouches of females of different hybrid index. Suppl. Fig. 4. Variation in proportion of abortive embryos among females in relation to shore height. Suppl. Fig. 5. Deviance residuals from the models in Supplement Table 3 in relation to transect location. Suppl. Fig. 6. The relationship between heterozygosity and hybrid index [file JEB-33-342-s001.pdf]
